# National cancer database analysis of outcomes in pediatric glioblastoma

**DOI:** 10.1002/cam4.1404

**Published:** 2018-03-13

**Authors:** Meng Liu, Jigisha P. Thakkar, Catherine R. Garcia, Therese A. Dolecek, Lars M. Wagner, Emily Van Meter Dressler, John L. Villano

**Affiliations:** ^1^ Division of Cancer Biostatistics University of Kentucky Lexington Kentucky; ^2^ Markey Cancer Center University of Kentucky Lexington Kentucky; ^3^ Department of Neurology University of Kentucky Lexington Kentucky; ^4^ Division of Epidemiology and Biostatistics and Institute for Health Research and Policy School of Public Health University of Illinois at Chicago Chicago Illinois; ^5^ Department of Pediatric Hematology and Oncology University of Kentucky Lexington Kentucky; ^6^ Department of Medicine University of Kentucky Lexington Kentucky; ^7^ Department of Biostatistical Sciences School of Medicine Wake Forest Baptist Health Winston Salem North Carolina; ^8^ Department of Neurosurgery University of Kentucky Lexington Kentucky

**Keywords:** Outcomes, pediatric glioblastoma, survival, treatment

## Abstract

Glioblastoma in children is an aggressive disease with no defined standard therapy. We evaluated hospital‐based demographic and survival patterns obtained through the National Cancer Database to better characterize children with glioblastoma. Our study identified 1173 patients from 0 to 19 years of age between 1998 and 2011. Comparisons were made among demographics, clinical characteristics, treatment, and survival variables. Fifty‐four percent of patients were over 10 years of age. Approximately 80% of patients underwent either partial or complete resection. Adjuvant therapy was used variably, and its use increased with patient age. Forty‐eight percent of patients received the combination of surgery, radiation, and chemotherapy, and 4% did not receive any treatment. As expected, patients ≤5 years of age had better 5‐year survival than those ages 6–10 (*P* = 0.01) or 11–19 years (*P* = 0.0077). Other factors associated with poor survival included black race and central tumor location. Better outcomes were associated with treatment that included surgery, radiotherapy, and chemotherapy compared to any other treatment combinations. Radiotherapy had no impact on survival in the 0 to 10‐year‐old age group, but was associated with improved survival for patients 11–19 years. We report an extensive demographic and survival analysis of pediatric glioblastoma. The observed differences likely reflect variances in tumor biology and likelihood of treatment receipt. Improved survival was associated with the use of surgery, radiotherapy, and chemotherapy. Radiation therapy was not associated with survival in patients younger than 10 years of age.

## Introduction

Brain and central nervous system tumors are one of the leading causes of cancer death in children 1, 2, 3. Pediatric glioblastoma (pGBM) is an aggressive high‐grade glioma associated with well‐established genetic subtypes with varying incidence, location, and outcomes based on general age categories 4. Younger children (<5 years of age) are generally associated with better prognosis and are associated with lesser number of mutations 5. In older children, H3F3A mutations at K27 occur and are associated with a marked dismal prognosis 5, 6. H3 K27‐mutant tumors are predominantly seen in midline locations (thalamus, pons, and upper spinal cord), and all other genetic variants are most commonly located in hemispheric locations 6. In adolescents, IDH mutant and giant cell glioblastoma (GBM) predominate and primarily affect hemispheric locations. These subtypes have biologic, prognostic, and growth pattern differences that make pGBM markedly different from their adult counterpart 6, 7.

Optimal therapy for pGBM is not well defined, maximum safe resection is performed when feasible, while the use of radiotherapy is routine for children over 3 years of age. Radiation is often deferred in infants due to the potential for injury to the developing brain. Instead, chemotherapy is often employed in an attempt to delay the need for radiation. In older patients, the use of chemotherapy following radiation is common, given the consistently disappointing survival rates with various regimens employed over the past few decades 8, 9, particularly temozolomide, with a 3‐year event‐free survival of 7% in a large cooperative study 8.

We analyzed the National Cancer Database (NCDB), which provides broad and detailed information on tumor characteristics, general treatment information, and survival outcome data, to characterize demographics, outcomes, and patterns of care of glioblastoma in pediatric patients.

## Methods

We performed an analysis of the NCDB, the largest cancer database in the world 10, using the 2011 participant user file for cases of glioblastoma in patients between 0 and 19 years of age. This participant user file contains data from 1998 to 2011 from over 1500 Commission on Cancer (CoC) accredited hospitals. NCDB is a hospital‐based registry from a coalition between the American College of Surgeon's Commission on Cancer and the American Cancer Society that is available after proposal approval for clinical investigators at CoC accredited cancer programs.

NCDB provides a large‐scale look at the patterns of care for cancer and has been used extensively in the study of several types of cancer 11, 12, 13 and in neuro‐oncology 14, 15. As patients with glioblastoma require specialized care, essentially all of the cases are expected to receive treatment in CoC accredited hospitals, making NCDB the most suitable registry to analyze patterns of care in this population. Its limitations rely on the retrospective nature of the data, the lack of specific chemotherapy drugs used, and inherent limitations of registry data for evaluating patient outcomes 16.

A total of 1173 patients were identified with diagnosis of glioblastoma, using the International Classification of Diseases for Oncology (ICD‐O3) histology codes 9440, 9441, and 9442 and tumor sites including brain stem, cerebellum, cerebrum, ventricle, brain not otherwise specified (NOS), and spinal cord (C70.0‐C72.9, C75.1‐C75.3). As available in NCDB, data variables are defined according to the Facility Oncology Registry Data Standards (FORDS) manual.

Patients were divided by age groups (0–5; 6–10; 11–19) determined by established tumor biological differences and previous reports 5, 6. Comparisons were made between age groups, gender, race, Hispanic origins, insurance (Not Insured; Private Insurance/Managed Care; Medicaid; Medicare/Other; Unknown), median household income (divided as: <$30,000; $30,000–$34,999; $35,000–$45,000; >$46,000), educational level, dwelling region, primary anatomical site (hemispheric [brain lobes], central location [ventricles, brain stem, and cerebellum], and others [spinal cord and brain not otherwise specified]), histology groups, and treatment modality (radiation, chemotherapy, and surgery; radiation and chemotherapy; radiation and surgery; chemotherapy and surgery; chemotherapy only; surgery only; radiotherapy only; none; and others). The others group was created by including patients who received either: none, one, or more treatment modalities, but are unknown for other therapies. Due to low frequencies and to ensure the reliability of the analysis, chemotherapy only, radiation only, and chemotherapy and surgery were included in the others group for multivariate analyses.

Dwelling region, educational level, and insurance status were derived from the patient's residential zip code at the time of diagnosis from the 2000 US Census data. Dwelling region was further categorized by comparing the patient's residential state and county Federal Information Processing Standard (FIPS) code at diagnosis to the 2003 Rural–Urban Continuum Codes as developed by the US Department of Agriculture Economic Research Service. Metropolitan region was defined as counties with a population of 250,000 or more; urban region had a population of 2500 to less than 250,000; rural region had a population of less than 2500 inhabitants.

Survival was defined as time from diagnosis until death due to all causes. Survival analysis was based on the years 1998–2006 due to the NCDB 5‐year lag in reporting survival data. Survival patterns were assessed using Kaplan–Meier survival estimates calculated for each demographic criteria and treatment plan. Cox proportional hazards models were employed to assess risk factor for mortality. Hazard ratio was defined as the average risk of death over the time period presented, due to the long‐term follow‐up. The level of statistical significance was set at 0.05 for all tests conducted, and all analyses were performed with SAS software version 9.4 (SAS Statistical Institute, Cary, NC).

## Results

### Demographics

We identified 1173 patients with from age 0 to 19 years from 1998 to 2011 in the NCDB, representing 1.15% of the total cases of glioblastoma (*N* = 101,846). Fifty‐three percent of the cases were diagnosed after 2005, with the highest percentage of patients diagnosed in 2011 (9.89%). Males had higher overall occurrence as compared to females (56% vs. 44%). Age group distribution was 21.5%, 24.5%, and 54.0% in the 0 to 5‐, 6 to 10‐, and 11 to 19‐year‐old age groups (Table 1). The majority of tumors were seen in whites and non‐Hispanics as compared to any other race. The most common anatomic location was hemispheric (58.49%), followed by others (22.75%), and central location (18.76%). The frequency of central location tumors was significantly lower with increasing age group (*P* = <0.0001). We found no significant difference between Hispanic origins, years of diagnosis, or education status between age groups.

**Table 1:** Summary of patient characteristics and demographics by age group in pediatric glioblastoma, 1998–2011

Summary of patient characteristics and demographics by age group, 1998–2011				
	0–5 *N* = 252 (21.48%)	6–10 *N* = 287 (24.47%)	11–19 634 (54.05%)	*P*‐value
Gender				
Male	123 (48.81%)	161 (56.10%)	374 (58.99%)	0.0225
Female	129 (51.19%)	126 (43.90%)	260 (41.01%)	
Race				
White	191 (75.79%)	213 (74.22%)	481 (75.87%)	0.9644
Black	37 (14.68%)	51 (17.77%)	99 (15.62%)	
Others	16 (6.40%)	16 (5.57%)	37 (5.84%)	
Unknown	8 (3.17%)	7 (2.44%)	17 (2.68%)	
Region				
Metro	198 (78.57%)	221 (77.00%)	478 (75.39%)	0.0814
Urban/Rural	35 (13.89%)	57 (19.86%)	119 (18.77%)	
Unknown	19 (7.54%)	9 (3.14%)	37 (5.84%)	
Primary site				
Hemispheric	121 (48.02%)	148 (51.57%)	417 (65.77%)	<0.0001
Central location	76 (30.16%)	77 (26.83%)	67 (10.57%)	
Others	55 (21.83%)	62 (21.60%)	150 (23.66%)	
Radiation				
Yes	118 (46.83%)	227 (79.09%)	499 (78.71%)	<0.0001
No	126 (52.77%)	58 (20.21%)	131 (20.66%)	
Unknown	1 (0.40%)	2 (0.70%)	4 (0.63%)	
Chemotherapy				
Yes	135 (53.57%)	181 (63.07%)	448 (70.66%)	<0.0001
No	109 (43.25%)	93 (32.40%)	162 (25.55%)	
Unknown	8 (3.17%)	13 (4.53%)	24 (3.79%)	
Surgical procedure of the primary site				
Resection^a^	195 (77.38%)	215 (74.91%)	527 (83.12%)	0.0071
No	57 (22.62%)	72 (25.09%)	106 (16.75%)	
Unknown	0	0	1 (0.16%)	
Treatment combination				
RT+CT+Surgery	63 (25%)	137 (47.74%)	363 (57.26%)	<0.0001
RT+CT	20 (7.94%)	34 (11.85%)	60 (9.46%)	
RT+Surgery	16 (6.35%)	28 (9.76%)	48 (7.57%)	
Surgery Only	62 (25%)	32 (11.15%)	73 (11.51%)	
None	15 (5.95%)	12 (4.18%)	21 (3.31%)	
Others	76 (30.16%)	44 (15.33%)	69 (10.88%)	
Income				
<$30,000	53 (21.03%)	46 (16.03%)	77 (12.15%)	0.0267
$30,000–$34,999	40 (15.87%)	43 (14.98%)	129 (20.35%)	
$35,000–$45,999	58 (23.02%)	84 (29.27%)	168 (26.50%)	
>$46,000	86 (34.13%)	103 (35.89%)	231 (36.44%)	
Unknown	15 (5.95%)	11 (3.83%)	29 (4.57%)	

RT, Radiotherapy; CT, Chemotherapy.

a Includes partial, gross, and total resection.

Around eighty percent of the patients received some form of surgery (gross, total, or partial resection). Over 65% received some form of combination therapy with radiation and/or chemotherapy. Four percent of the cases did not receive any form of treatment. The frequency by treatment modality was: combination of surgery, radiotherapy, and chemotherapy (48%); surgery only (14.2%); radiation and chemotherapy (9.72%); radiation and surgery (7.84%); chemotherapy and surgery (6.05%); radiation only (4.77%); chemotherapy alone (0.93%); others (4.34%); and none (4.09%).

**Figure 1:**
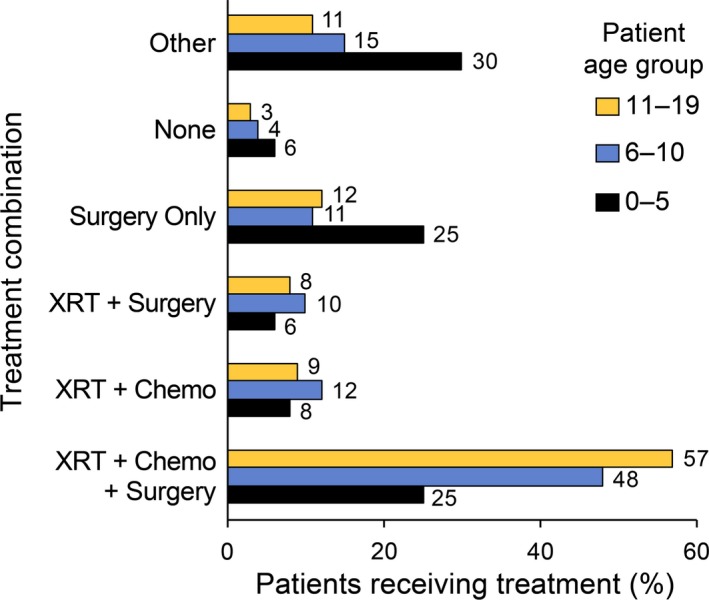
Receipt of treatment combination in different pediatric age groups from NCDB, 1998–2011. *X*‐axis: percentage of pediatric glioblastoma cases from NCDB, 1998–2011. *Y*‐axis: different treatment combinations received. Abbreviations: RT, radiation; CT, Chemotherapy.

We found a significant difference in treatment distribution by age group (Fig. 1). Patients younger than 5 years of age were more likely to receive others (30.16%), or surgery alone (24.60%), while combinatorial treatment was preferred in older patients. In the 6 to 10‐ and 11 to 19‐year‐old age group, 48% and 57%, respectively, received chemotherapy, radiation and surgery, followed by either radiation and chemotherapy, or radiation and surgery.

The majority of patients had Private Insurance or Managed Care (56.48%), followed by Medicaid (26.34%), Unknown (6.39%), Not Insured (4.77%), and Medicare/Other (4.01%). Insurance rates differed while comparing age groups (*P* = 0.008), probably related to Medicaid, and Medicare proportions. The majority of the patients lived in metropolitan areas (76.47%), with over 60% of patients with a median income greater than $35,000/year. Income was statistically different between age groups (*P* = 0.008).

### Survival analysis

**Figure 2:**
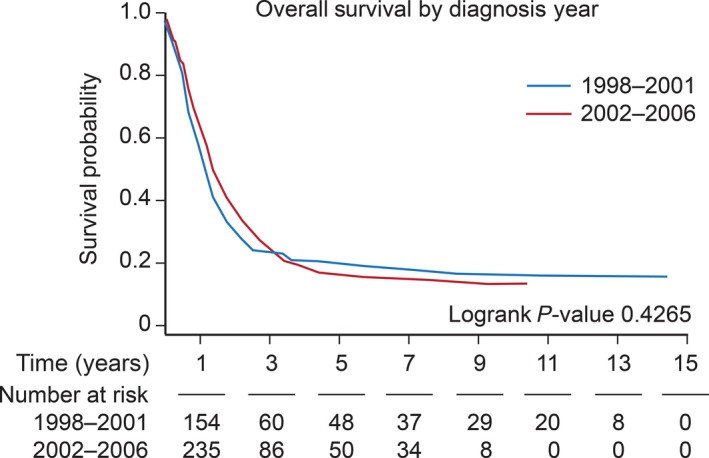
Kaplan–Meier survival by years of diagnosis 1998–2001 and 2002–2006. *X*‐axis: time in years. *Y*‐axis: survival probability.

The overall median survival was 15 months. Overall one‐ and five‐year survival after diagnosis was 58% and 17%, respectively, in children diagnosed with glioblastoma from 1998 to 2006. The one‐ and five‐year survival rates for the three age groups were 52.2% and 29.8% (0–5 years old), 51.2% and 14.1% (6–10 years old), and 64.3% and 13.2% (11–19 years old). We compared hazard ratios between the years 1998–2001 (1.09 CI: 0.95, 1.27) and 2002–2006 (1.31 CI: 1.21, 1.53) and found no statistical difference (*P* = 0.4265) (Fig. 2). Age groups over 5 years of age were identified as risk factors for mortality in the Cox proportional hazard model; 6 to 10‐year‐old group (HR 1.408 CI: 1.069–1.854, *P* = 0.01) and 11 to 19‐year‐old group (HR 1.406 CI: 1.094–1.806, *P* = 0.0077) (Table 2). Black race was associated with poorer survival outcomes compared to white race. Tumor location was significantly associated with survival, showing worse outcomes in patients with tumors with central location than hemispheric location.

**Table 2:** Cox proportional hazard model for independent risk factors of survival in pediatric glioblastoma

Cox proportional hazard model for independent risk factors of survival			
	Hazard ratio	95% Confidence interval	*P*‐value
Age groups			
0–5	Ref	–	
6–10	1.41	1.069–1.854	0.015
11–19	1.41	1.094–1.806	0.008
Race			
White	Ref	–	
Black	1.29	1.017–1.656	0.036
Other	1.04	0.718–1.521	0.8181
Income			
<$30,000	1.173	0.895–1.536	0.2471
$30,000–$34,999	1.492	1.171–1.9	0.0012
$35,000–$45,999	1.198	0.966–1.487	0.0996
>$46,000	Ref	–	–
Primary Site			
Hemispheric	Ref	–	
Central location	1.467	1.153–1.868	0.0018
Others	0.986	0.793–1.227	0.9022
Treatment combination			
None	Ref	–	–
RT+CT+Surgery	0.53	0.341–0.824	0.0048
RT+CT	0.883	0.537–1.454	0.6257
RT+Surgery	0.727	0.435–1.215	0.2232
Surgery alone	0.905	0.564–1.451	0.6773
Other	0.62	0.387–0.995	0.0476

RT, Radiotherapy; CT, Chemotherapy.

**Figure 3:**
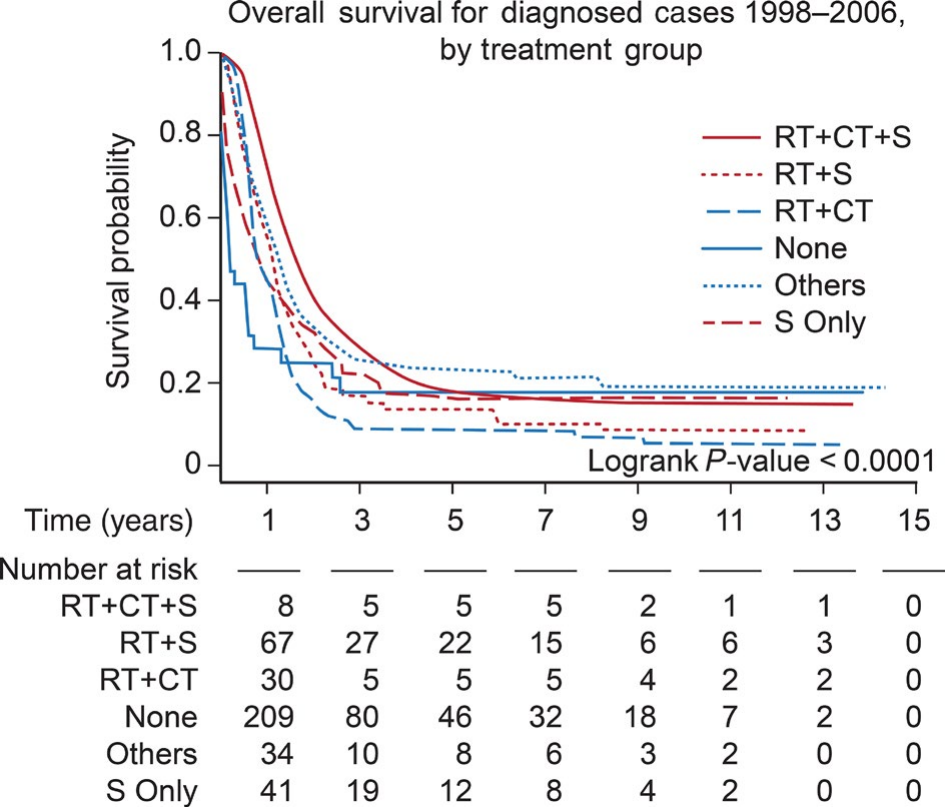
Kaplan–Meier survival by receipt of treatment in pediatric glioblastoma from 1998 to 2006. *X*‐axis: time in years. *Y*‐axis: survival probability. Abbreviations: RT, radiation; CT, chemotherapy; S, surgery.

**Figure 4:**
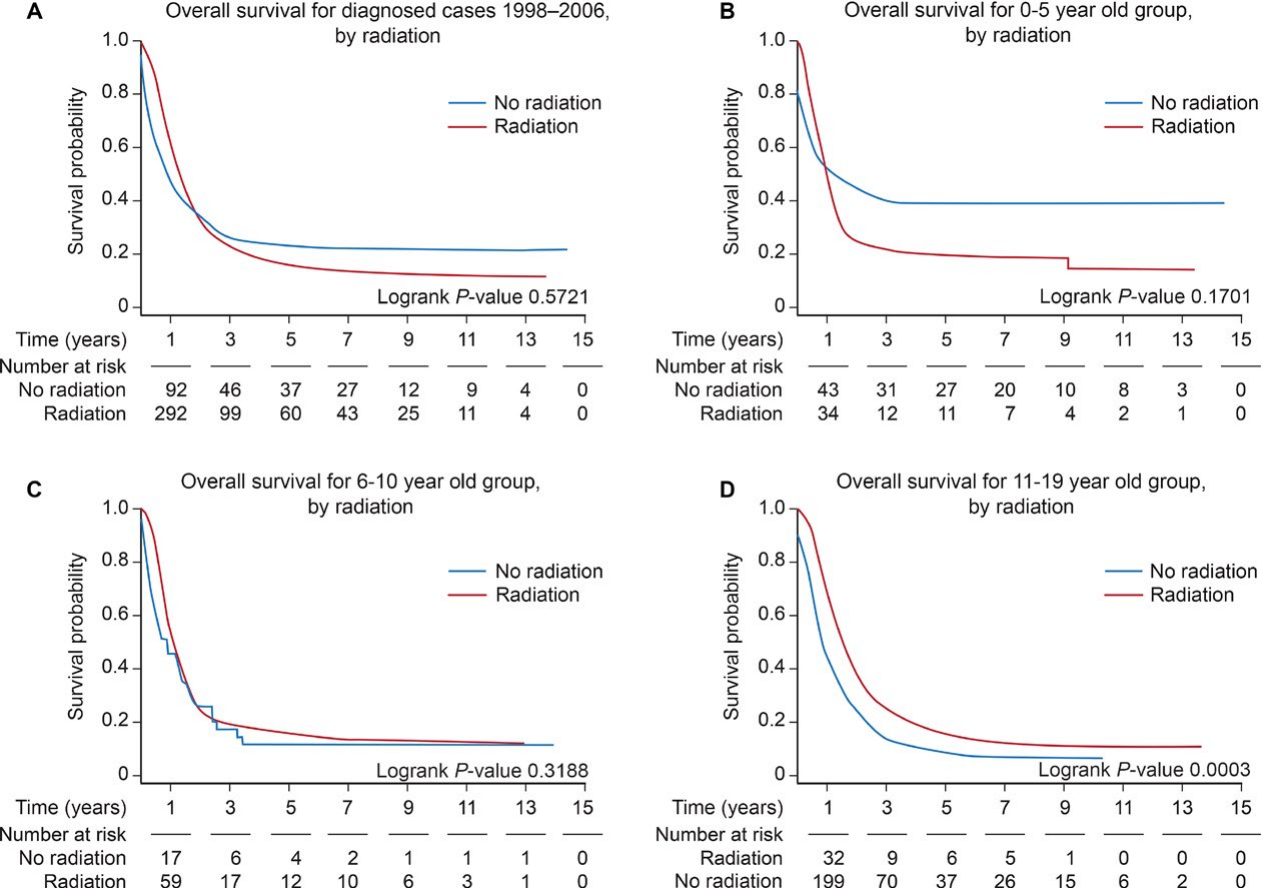
Kaplan–Meier survival in patients receiving radiation in the 0 to 5‐, 5 to 10‐, and 11 to 19‐year‐old age groups from 1998 to 2006. *X*‐axis: time in years. *Y*‐axis: survival probability. (A) Kaplan–Meier survival in all pediatric patients receiving radiation. (B) Kaplan–Meier survival in the 0 to 5‐year‐old age group. (C) Kaplan–Meier survival in the 6 to 10‐year‐old age group. (D) Kaplan–Meier survival in the 11 to 19‐year‐old age group.

Treatment received was associated with survival (Fig. 3), particularly surgery, chemotherapy, and combination of surgery, chemotherapy, and radiation. The usage of radiation demonstrated no impact on survival (Fig. 4A). We performed a subgroup analysis on the association of radiation with survival by age group and found no association in patients younger than 10 years of age (Fig. 4B and C). However, a positive association was found in older patients (11 to 19‐year‐old age group) (Fig. 4D). Incomes lower than $46,000 annually were associated with increased mortality. Insurance status, educational level, dwelling region, and tumor histology showed no association with survival.

## Discussion

GBM is a devastating diagnosis at any age and never more so in children. Although GBM is a disease known to affect adults, it remains deadly cancer in children. Our analysis details the largest published series of patients with pGBM with a broad representation of the United States demographics. Previous analyses on pGBM have had fewer cases or have been combined with other high‐grade gliomas 1, 17.

The five‐year survival in our analysis for pGBM was 17%, much lower than described for pediatric HGG 1, with outcomes significantly worse than for any of the common pediatric cancers such as leukemia, osteosarcomas, and neuroblastomas 1. Pediatric brain tumors are generally associated with long‐term survival 1, 15. Our analysis demonstrated that the mortality of pGBM is greater than any other primary brain tumor, which includes our previous NCDB investigation within the same years of analysis for medulloblastoma 15.

Demographic patterns were similar to adult GBM with males and whites representing the majority of cases 1, 3, 18, 19, 20. Blacks were associated with poorer survival outcomes compared to whites, which has also been found in population‐based studies for adults with primary brain tumors and in many other cancers 21. We hypothesize these findings are associated with sociodemographic factors and access to neuro‐oncological care, as described by other studies 22, as there has not been a race‐based genetic or molecular correlation in GBM identified. In addition, incomes lower than $46,000 was associated with higher mortality.

Younger age was associated with better survival, as described in smaller cohorts of high‐grade gliomas 8, 23, 24, 25, which is generally associated with differences in tumor biology 6, 26. Hemispheric lesions were more common and had a higher frequency in older children. Although more frequent with an early age, centrally located tumors had higher mortality as compared to hemispheric location. This is comparable to other descriptions in the literature, as centrally located tumors are very aggressive and associated with histone 3 mutations, particularly H3F3A mutations at K27 18, and is classified as H3 mutant (H3 K27M) diffuse midline glioma 4. Prior analysis of the surveillance, epidemiology, and end results database found no significant difference in survival patterns in pediatric glioblastoma while separating brainstem tumors from the rest of the anatomical locations 17.

Pediatric GBMs have a greater frequency of cancer predisposition syndromes including germline mutations in TP53 (Li‐Fraumeni syndrome) and the mismatch repair (MMR) genes (biallelic MMR deficiency syndrome [bMMRD]), which recently demonstrated significant response to immune checkpoint inhibitors 27, which points to a subset of pGBM anticipated to improve over time. IDH1 mutation is associated with favorable prognosis in adult patients, but is not commonly seen in pediatric patients younger than 10 years of age 28, 29, and although some subtypes of pGBM, particularly H3 K27M, show a similar expression patterns to the adult IDH mutant 6, 30, they do not share its positive survival outcomes 29, 31. This may point to underlying genetics as well as age‐related metabolomics differences 32, which may also explain differences in treatment response between adult and pediatric patients.

Reviewing a nationwide pattern of care provides a reference with the goal of assessing the range of treatment. Any form of combinatorial therapy was associated with better survival regardless of age. The highest survival benefit was seen in patients receiving chemotherapy, radiotherapy, and surgery as first‐line treatments, which is in keeping with the literature 33, 34. This multi‐modality approach is usually attempted in children older than 3–5 years of age 35. In younger children, chemotherapy alone, and radiation sparing treatment, is preferred to prevent the adverse effects of radiation to the developing brain 23, 24, with a relatively safe profile as first‐line treatment 36, 37. Chemotherapy was used in over 60% of patients as first course of treatment demonstrating positive survival benefits. It is important to note that salvage chemotherapy is not captured with NCDB. The usage of chemotherapy in combination with surgery and radiation in pediatric patients has shown limited survival benefits in clinical trials combining different grades of gliomas 8, 36, 38, 39. In distinction in adults, the practice of combining different grade of gliomas has given away to treatments based on underlying genetic biology or tumor grade.

We found age‐related benefits in the usage of radiation, only becoming beneficial in patients older than 10 years of age. This could be attributed to a higher frequency of centrally located tumors in patients younger than 10 years of age, and the low usage of radiotherapy in children younger than 5 years of age. As previously stated, H3F3A mutations are frequent in centrally located tumors 6, these mutations largely overlap with TP53 mutations 40, that have been linked with a diminished response to radiation 41, 42, 43. The optimal dose of radiation in pGBM remains undetermined. Information regarding specific radiation dose and schedule is not available in the NCDB database, and survival may vary somewhat according to these details of administration 34.

The main limitations of our analysis are due to the retrospective and observational nature of the NCDB database. Molecular and pathology studies are not included in our model, along with the indications for receipt of a particular therapy. NCDB's data collection does not allow incidence rates to be estimated, but provides detailed clinical descriptions of tumors at diagnosis, treatment information, and survival outcome data. The broad coverage and large numbers of cases included in the hospital‐based NCDB data approximate the population‐based registries for descriptive statistics.

In summary, our use of NCDB database provides a larger sample size than previously published and allows a comprehensive overview on statistics, treatment patterns, and survival.

## Conflict of Interest

JPT, EVD, and JLV were supported by the National Cancer Institute (R03CA156561), and EVD and ML were members of the Biostatistics and Bioinformatics Shared Resource Facility of the University of Kentucky Markey Cancer Center (P30CA177558). The funding source had no role in writing the manuscript or the decision to submit for publication.
